# Suture pattern formation in ammonites and the unknown rear mantle structure

**DOI:** 10.1038/srep33689

**Published:** 2016-09-19

**Authors:** Shinya Inoue, Shigeru Kondo

**Affiliations:** 1 Graduate School of Frontier Bioscience, Osaka University, Yamada 1-3, Suita, Osaka 565-0871, Japan; 2 Hokkaido University Shuma-no-kai, 354 Clubs and Societies Building, Kita 17, Nishi 12, Kita-ku, Sapporo, Hokkaido 060-0808, Japan

## Abstract

Ammonite shells have complex patterns of suture lines that vary across species. The lines are formed at the intersection of the outer shell wall and the septa. The wavy septa can form if the rear mantle of the ammonite, which functions as the template, has a complex shape. Previous hypotheses assumed that the rear mantle is like a flexible membrane that can be folded by some physical force. The elucidation of the mechanism of septa formation requires that the detailed shape of the septa should be known. We developed a new protocol of X-ray micro-computed tomography (CT) and obtained high-resolution three-dimensional (3D) images of the septa of the Upper Cretaceous ammonite *Damesites* cf. *damesi*. The obtained image suggested that the wavy and branched structures of the rear mantle grew autonomously. We found that some extant sea slugs have branched structures and showed similar shape and growth sequence as those in fossils, suggesting that the mantle of molluscs basically has the potential to form branched projections. Based on the characteristics of the obtained 3D structure, we explain how ammonites might have formed the complex suture patterns.

Ammonite conchs are separated into small chambers by septa, which might contribute to reinforce the shell wall^1^,^2^,^3^,^4^ and control buoyancy^5^,^6^,^7^. The wavy line of intersection between the shell and septum is called the suture line, which is highly diverged among ammonite species; it is a useful index for taxonomical identification^8^,^9^,^10^,^11^. However, very little is known about the mechanism of how this complex and diverged pattern was generated.

Living *Nautilus* also have septa; they form these septa by using their round rear mantle as a template^12^. Since ammonites are related to *Nautilus*, the ammonite septa should also have been formed by using the rear mantle as a template. However, the formation of the complex suture pattern requires extensive deformation of the rear mantle. In the two previously proposed hypotheses, the rear mantle of ammonites was assumed to be flexible and could be passively folded by some external force into complex folds.

The tie-point model, the best-known hypothesis, assumes that the rear mantle adheres to the shell wall at many small points known as tie-points^13^,^14^,^15^,^16^. If hydrostatic pressure was applied from the apical side, the mantle would be pressed to move adorally, but the tie-point region would remain at the same position and eventually form an arch-like shape.

The other model is the viscous fingering model^17^,^18^,^19^,^20^. According to this model, when two fluids with different viscosity come in contact, the Saffman–Taylor instability is induced at the interface, generating finger-like shapes that are similar to the ammonite suture patterns. This model assumes that the Saffman–Taylor instability occurs on the surface of the rear mantle and in the fluid of the shell chamber. Although these models explain the formation of the suture pattern via a simple method, both have a problem. These models are not based on the phenomena found in extant organisms. Therefore, validating these models is difficult.

Hammer presented another idea in 1999^21^. His model does not involve any specific physical method, but simply assumes that the peripheral region of the rear mantle invaginates to form the folding of the septa.

Previous models mainly focused on the two-dimensional (2D) suture pattern and considered the behaviour of the marginal region of the ammonite mantle. However, because the suture lines are the intersection between the shell wall and septa, understanding how the three-dimensional (3D) structure of the septa is formed is necessary. Thus, we developed a method to obtain a super-high resolution 3D image of ammonite septa, and then compared the previous models with the obtained image.

## Results

### 3D imaging of ammonite septa

**Figure 1:**
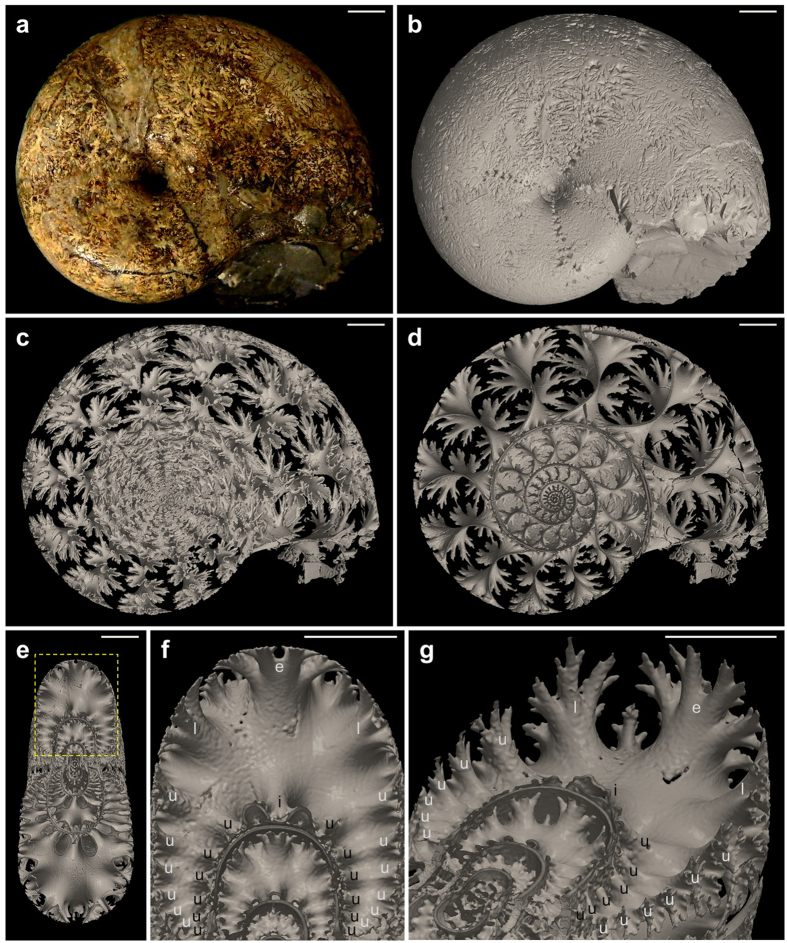
Micro-computed tomography (CT) images of *Damesites* cf. *damesi*. (**a**) Incident light photograph, left lateral view of NMA00802. (**b**) Micro-CT image of (**a**). (**c**) Three-dimensional data of the septa extracted from (**b**). (**d**) Median section of (**c**). (**e**) Transverse section of (**c**). (**f**) Magnified image of a yellow dotted square in (**e**). (**g**) Diagonally upward view of (**f**). e, external arm; l, lateral arm; u, umbilical arm; i, internal arm. Septal arms in the outer and inner regions are shown with white and black characters, respectively. Scale bars, 5 mm.

To determine how ammonites formed the complex suture patterns, we performed detailed 3D analysis of the ammonite shells. Various types of ammonite fossils that vary in mineral diagenesis are available. In some types of ammonite fossils, the shell chambers were filled with calcite; diagenesis then dissolved and eliminated the shell wall and septa, generating an empty space at their place. We analysed such samples by using micro-computed tomography (CT) and obtained almost the complete sequence of septal structure with the highest resolution (Supplementary Figs 1–6). The 3D image for specimen NMA00802, Upper Cretaceous ammonite *Damesites* cf. *damesi* (Ammonitina, Desmoceratidae) from Hokkaido, is shown in Fig. 1. In addition to the septa, other structures such as septal necks, siphuncle, and cameral sheets were visualised in high resolution (Supplementary Fig. 7). The septa appeared like repeated finger-like structures dividing at the edge (Fig. 1f,g). We named these finger-like structures as septal arms. To describe the detail of the septal structure, we referred these septal arms as external, lateral, umbilical, and internal arms, following the conventional terminology of suture patterns. The external arm was the largest and the most extensively divided. The lateral and umbilical arms were smaller and had lesser branches. Therefore, the density of the septal tips was almost uniform, resulting in the fractal appearance of the entire structure.

### Tubular structures in ammonite septa

**Figure 2:**
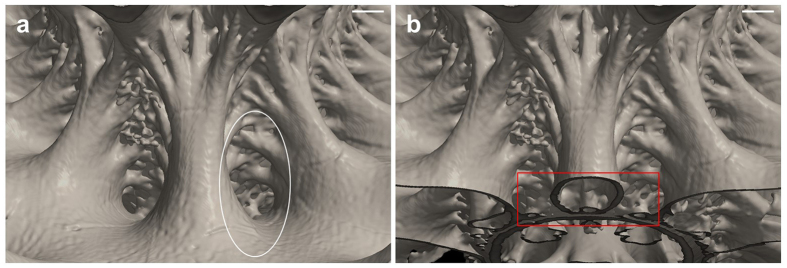
The tubular septal structures in the inner region of *Damesites cf*. *damesi*. (**a**) Inner region of the septum of NMA00802. The fine branches that are alternately located, highlight with a white circle. (**b**) Transverse section of the internal arm and small arms in (**a**), highlighted with a red square. Scale bars, 1 mm.

The margin of the arms occasionally joined with the previous septum as well as with the shell wall (Fig. 2). The number of septum-to-septum joints was low in the outer (ventral) region and high in the inner (dorsal) region. In the inner regions, the arm sections often formed a complete circle, indicating that they did not have any margin, but had a tubular structure, which is known as septal lobe in lytoceratid ammonites^22^ (Fig. 2). We believe that the formation of such a tubular structure might not be possible by the previous models for the following reasons. Since the viscous fingering effect occurred only in the quasi-2D field, such 3D structure should be beyond expectation of the model. Formation of the septal lobes by the tie-point mechanism requires an irregular condition. That is, tie-points need to occur at the inside of the rear mantle, but not at the edge. In addition, the tips of the tubular structures divide into many fine branches. The gaps of these branches are alternately filled with the fine branches of the adjacent arm (white circle in Fig. 2a). Formation of such a structure requires that the branches are formed before they touch the old septum.

To determine whether such a structure is common among ammonite species, we examined one more species, *Hauericeras* sp., belonging to the same family as that of *Damesites* cf. *damesi*, and two other species from different suborders, *Tetragonites* sp. (Litoceratina, Tetragonitidae) and *Hypophylloceras* sp. (Phylloceratina, Phylloceratidae), which confirmed the generality of this structure (Supplementary Fig. 8).

### Similarities between ammonite septa and sea slug cerata

Branched projections are not a rare structure, but rather commonly found in various animals; in particular, gastropod molluscs of the superfamily Dendronotoidea (a group of sea slugs) have a wide variety of branched projections known as cerata; their patterns resemble the suture lines (and naturally also the septa) of ammonoids (Supplementary Fig. 9). Because the cerata of sea slugs develop from the mantle, a similar mechanism can be assumed in ammonites.

**Figure 3:**
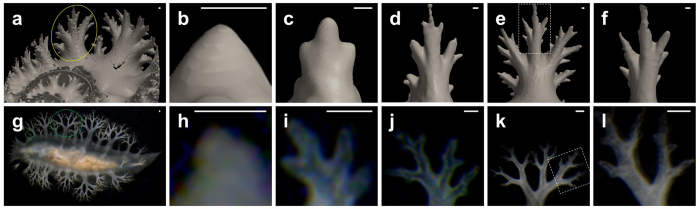
Comparison of the branching of the septa of *Damesites* cf. *damesi* and the cerata of *Tritoniopsis elegans*. (**a**) Diagonally upward view of a septum of NMA00802. (**b–e**) The branching process of the lateral arm, indicated with a yellow circle in (**a**). (**f**) Magnified image of a dotted square in (**e**). (**g**) Dorsal view of *Tritoniopsis elegans*. (**h–j**) The growth process of the cerata, indicated with a small green circle in (**g**). (**k**) A well-branched ceras, indicated with a large green circle. (**l**) Magnified image of a dotted square in (**k**). Scale bars, 0.2 mm.

**Figure 4:**
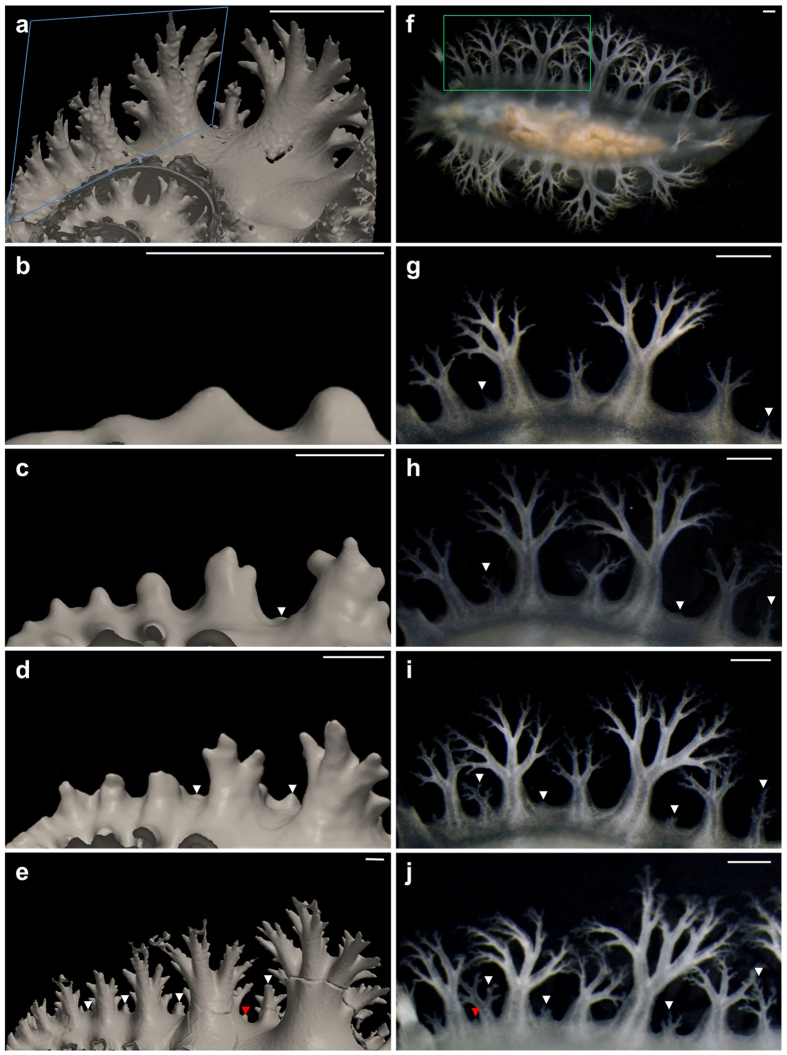
Comparison of the budding of the septa of *Damesites* cf. *damesi* and the cerata of *Tritoniopsis elegans*. (**a**) Diagonally upward view of a septa of NMA00802. (**b–e**) The budding process of the septal arms in the blue square of (**a**). 1^st^, 18^th^, 23^rd^, and 40^th^ septa of forty consecutive septa. (**f**) Dorsal view of *Tritoniopsis elegans*. (**g–**j) The budding process of the cerata in the green square of (**f**). Observations performed every three days. White and red arrowheads indicate new septal arms between the arms/cerata and adjacent to them, respectively. Scale bars, 1 mm.

Although living ammonites are not available, the micro-CT imaging allowed the observation of a series of septa that led to the growth of the mantle projection. When the growth sequence of sea slug cerata and ammonite septa were compared (Figs 3 and 4), the similarity between the two organs could be estimated.

The growth sequence of a lateral arm of *Damesites* cf. *damesi* is shown in Fig. 3b–e. When the organisms become older and larger, the septal folding becomes more complex by the repetitive branching. A part of the septal arm (Fig. 3f) is very similar to the entire structure of the younger arm (Fig. 3d), suggesting that the arm branching is a fractal structure. We could grow the sea slug *Tritoniopsis elegans* in a sea water tank and record how the shape of the cerata changed (Fig. 3h–j). A single ceras repetitively divided and formed a branched structure in the similar manner as that in the ammonite (Fig. 3h–j). A part of a large ceras (Fig. 3l) is similar to the entire ceras of a young sea slug (Fig. 3j), suggesting that the structure is fractal.

During the enlargement of the shell, small new arms appeared between the old and big arms. New septal arms appeared first at the most ventral region where the interval between the arms was the widest, and then gradually appeared in more lateral regions (Fig. 4b–e). This mode of arm insertion implies that the timing of the new arm insertion was dependent on the space without the arms (Hammer also pointed out this tendency and suggested the involvement of the lateral-inhibition mechanism^21^). A similar tendency was observed in the sea slug. The wider the spacing between the old cerata, the earlier was the appearance of the new ceras (Fig. 4g–j). Therefore, the growth process of the ammonite septa and sea slug cerata is very similar, and both might be controlled by similar mechanisms.

## Discussion

Our observations suggest that the rear mantle of ammonites might have self-grown projections whose shape and growth sequence resembled the cerata of sea slugs. The projections could form without the external physical method because sea slugs can form the dorsal projections autonomously in the absence of shells. This ‘cerata-septa model’ is close to the idea presented by Hammer (1999) and has some advantages compared to the other models. First, the cerata-septa model is based on existing biological phenomenon, which is more reasonable than postulating the phenomena not found in extant organisms. Branching is one of the common phenomena in organisms, especially in molluscs. Therefore, ammonites could have formed projections in a similar manner.

Second, the model can account for the variety of suture patterns among ammonite species and can be applicable to ammonoid species because the branching pattern of cerata varies extensively among the species (Supplementary Fig. 9). Comparing various ammonoid septa and sea slug cerata is necessary for regarding the cerata-septa model as a unified theory.

**Figure 5:**
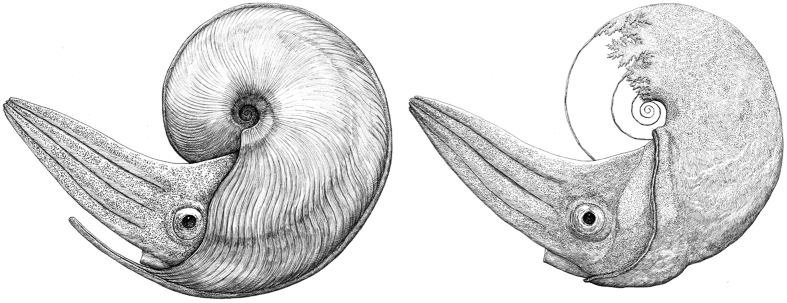
Reconstruction of the soft part of *Damesites* cf. *damesi*. Whole body (left) and soft part (right) of *Damesites* cf. *damesi* (illustrated by Takashi Oda).

Third, the cerata-septa model also considers the soft part of the ammonites (Fig. 5; Supplementary Fig. 10), which remains almost unknown^23^,^24^. If the rear mantle of ammonites undergoes suturing via a similar mechanism as that in sea slugs, experimentally inferring the nature of the ammonite soft part by using extant sea slug might become possible.

## Methods

### Ammonite specimen

Our observation of *Damesites* cf. *damesi* is based on the fossil specimen NMA00802 reposited at the Nakagawa Museum of Natural History, Hokkaido, Japan. This specimen was excavated from an outcrop (44.229°N, 142.005°E) exposed along a small tributary of the Chimeizawa River in Haboro district, Hokkaido, Japan. The fossil was preserved in a calcareous sandy mudstone nodule of the Haborogawa Formation, Yezo Group, with fine woody fragments. According to Hayakawa *et al.*^25^, the fossil-bearing horizon can be correlated with Santonian. In this study, we followed the classification of *Damesites* established by Nishimura *et al.*^26^, and NMA00802 can be referred to as *Damesites* cf. *damesi*, S-group morphotype. The following three additional ammonites are shown in Supplementary Fig. 2: *Tetragonites* sp. is based on NMA00803 occurring with NMA00802; *Hypophylloceras* sp. is based on NMA00811 from the Yezo Group (Upper Cretaceous) of Tomamae district, Hokkaido; *Hauericeras* sp. is based on NMA00812 from the Yezo Group (Upper Cretaceous) of Hokkaido; all these specimens are reposited at the Nakagawa Museum of Natural History. These specimens are selected by sight on the basis of the condition that the chambers are filled with calcite and the septa are completely dissolved.

### Micro-computed tomography

Fossils were scanned using ScanXmate-D150-S270 (Comscantecno) to obtain projection data, and the data, including 3D reconstruction data, were processed and visualised using Molcer Plus software (White Rabbit). The tube voltage was optimised to 151 kV. The reconstructed 3D images consisted of cubic voxels, and their resolution was 30.71, 29.14, 22.37, and 39.92 μm for *Damesites* cf. *damesi*, *Tetragonites* sp., *Hypophylloceras* sp., and *Hauericeras* sp., respectively. A continuous domain of the shell and septum was extracted for analyses by manually fixing minor discontinuities between voxels that were probably caused by tiny cracks or noise. A domain of the outer shell was also manually selected and masked in order to observe clearly the structure of the septum (Figs 3 and 4).

### Observation of a sea slug

*Tritoniopsis elegans* was collected from Hachijo-jima, Tokyo, Japan. It was maintained in artificial seawater at 23–24 °C and fed soft coral, *Cladiella digitulata*. Subsequently, it was weakly anesthetised with artificial seawater containing 100 mM MgCl_2_ and covered with a 50 × 70-mm cover glass (Matsunami Glass); it was viewed and imaged using a Leica MZ16 FA stereomicroscope equipped with a DFC300FXR2 digital camera (Leica) every three days.

## Additional Information

**How to cite this article**: Inoue, S. and Kondo, S. Suture pattern formation in ammonites and the unknown rear mantle structure. *Sci. Rep.* **6**, 33689; doi: 10.1038/srep33689 (2016).

## Supplementary Material

Supplementary Information
