# Supplementary material for: Suture pattern formation in ammonites and the unknown rear mantle structure
Source: Sci Rep. 2016 Sep 19;6:33689. doi: 10.1038/srep33689 (PMC5027383; doi:10.1038/srep33689)
Supplement: Supplementary Information [file srep33689-s1.pdf]

Supplementary information for Suture pattern formation in ammonites and the unknown rear mantle structure

Shin-ya Inoue[1, 2*] and Shigeru Kondo[1]

[1]Graduate School of Frontier Bioscience, Osaka University
Yamada 1-3, Suita, Osaka 565-0871, Japan

[2]Hokkaido University Shuma-no-kai
354 Clubs and Societies Building, Kita 17, Nishi 12,
Kita-ku, Sapporo, Hokkaido 060-0808, Japan

*Correspondence to Shin-ya Inoue (inoue.s.shuma@gmail.com, shin-ya@fbs.osaka-u.ac.jp)

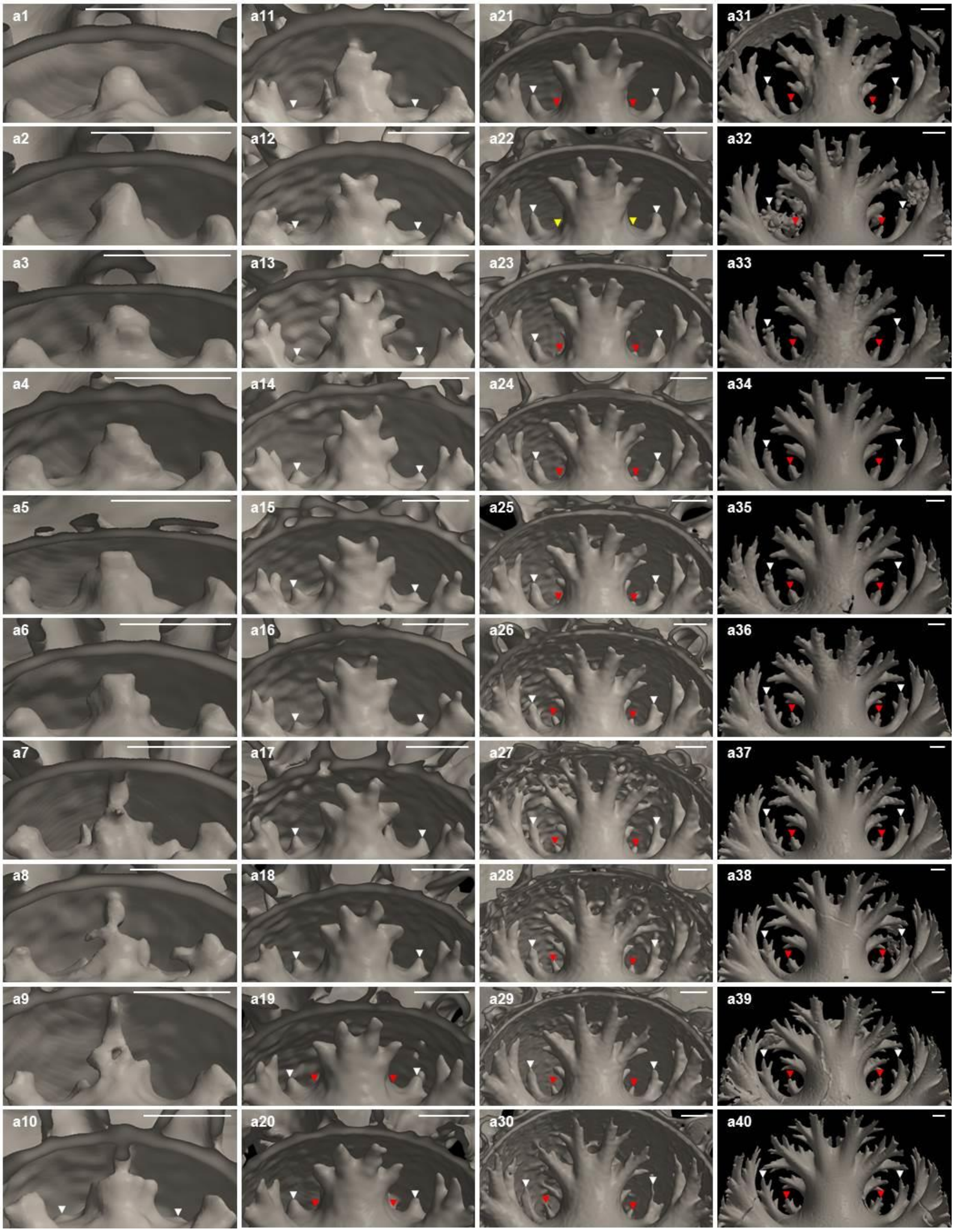

Supplementary Figure 1 | Three-dimensional data of forty consecutive external arms of *Damesites* cf. *damesi*. a1–40, New septal arms are indicated with arrowheads as in Figure 4. Yellow arrowheads indicate hidden septal arms. Scale bars, 1 mm.

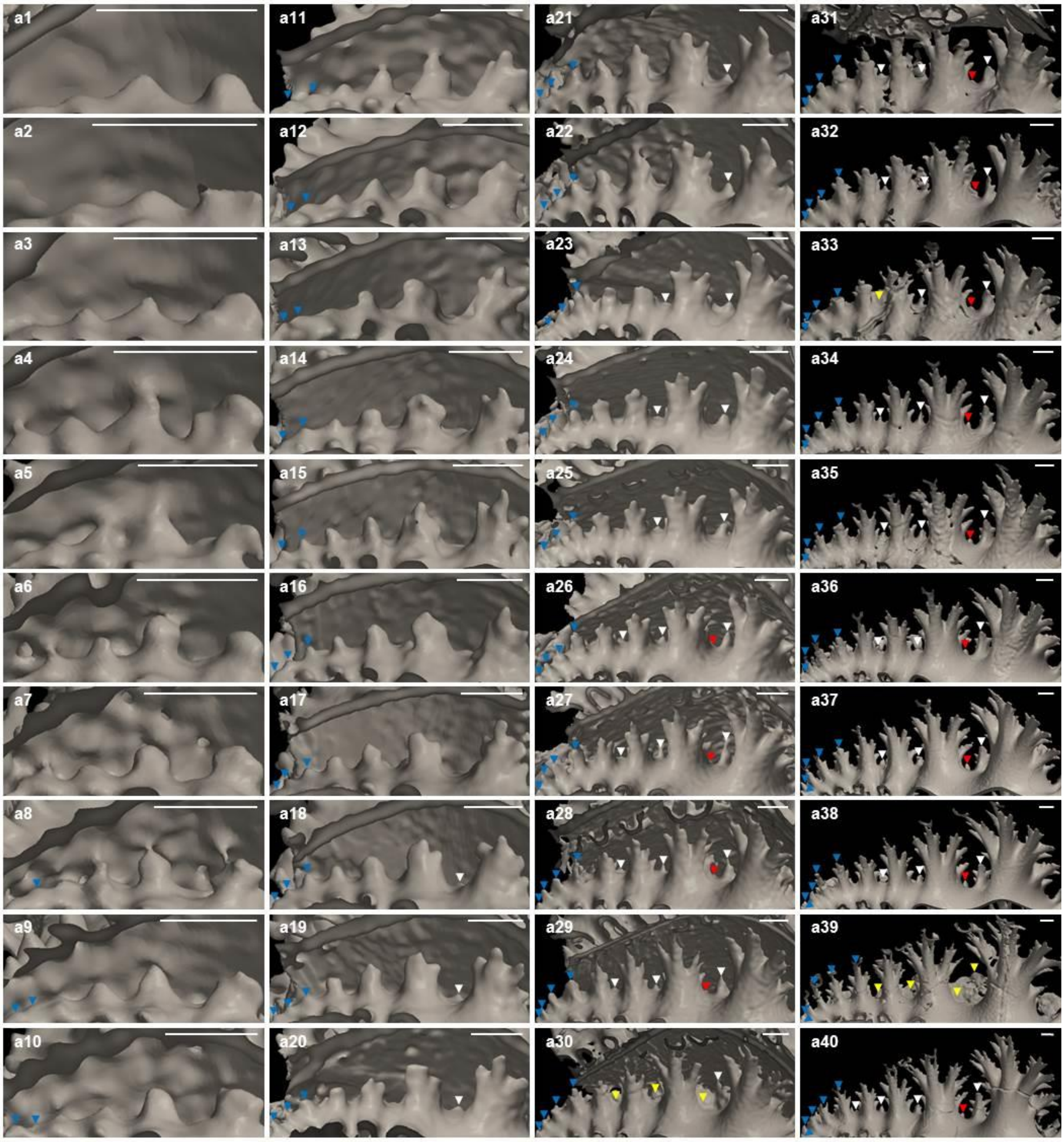

Supplementary Figure 2 | Three-dimensional data of forty consecutive lateral and umbilical arms of *Damesites* cf. *damesi* in the outer right regions. a1–40, New septal arms are indicated with arrowheads as in Figure 4. Blue and yellow arrowheads indicate the new septal arms at the distal site and hidden septal arms, respectively. Scale bars, 1 mm.

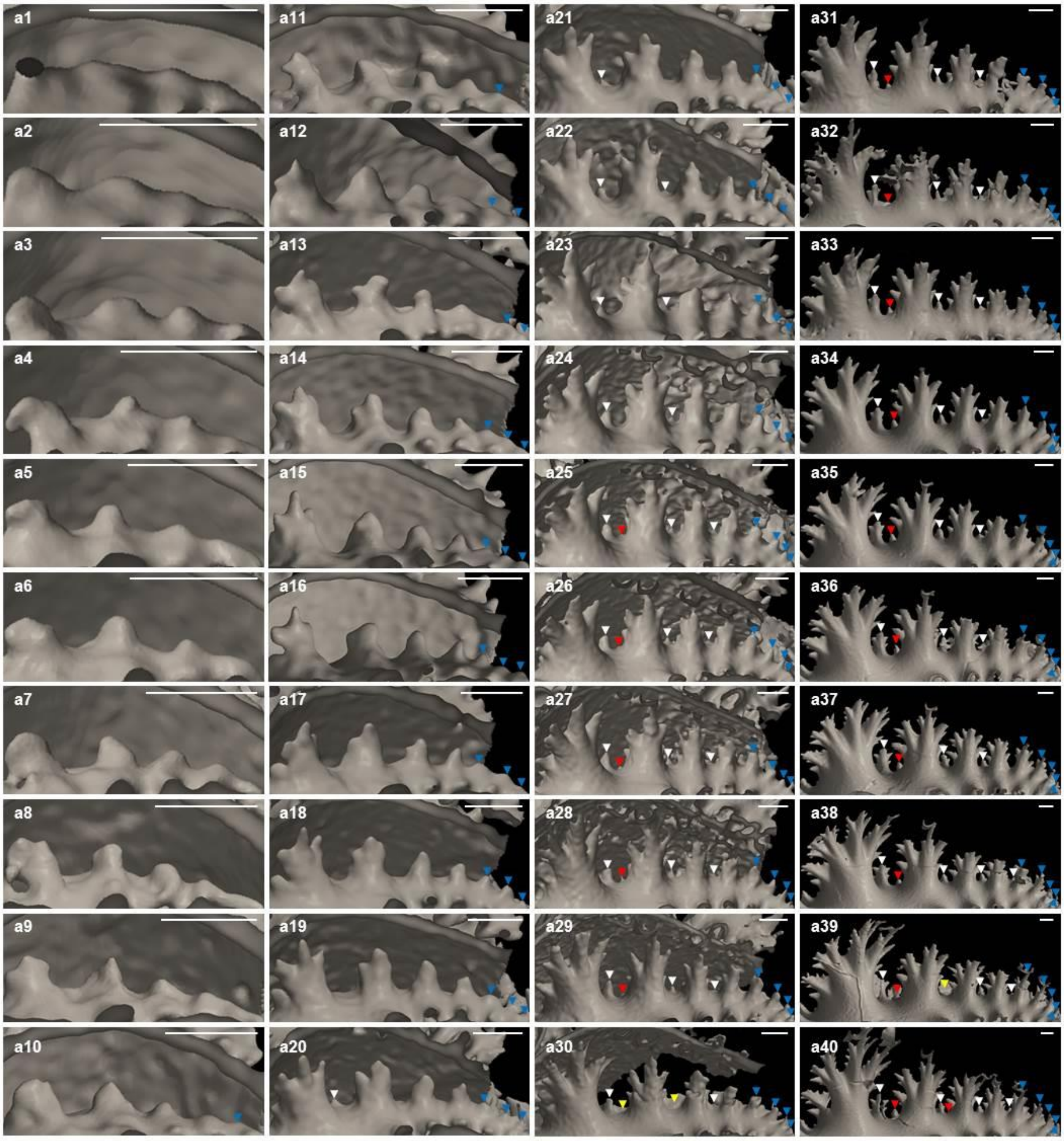

Supplementary Figure 3 | Three-dimensional data of forty consecutive lateral and umbilical arms of *Damesites* cf. *damesi* in the outer left regions. a1–40, New septal arms are indicated with arrowheads as in Figure 4. Blue and yellow arrowheads indicate the new septal arms at the distal site and hidden septal arms, respectively. Scale bars, 1 mm.

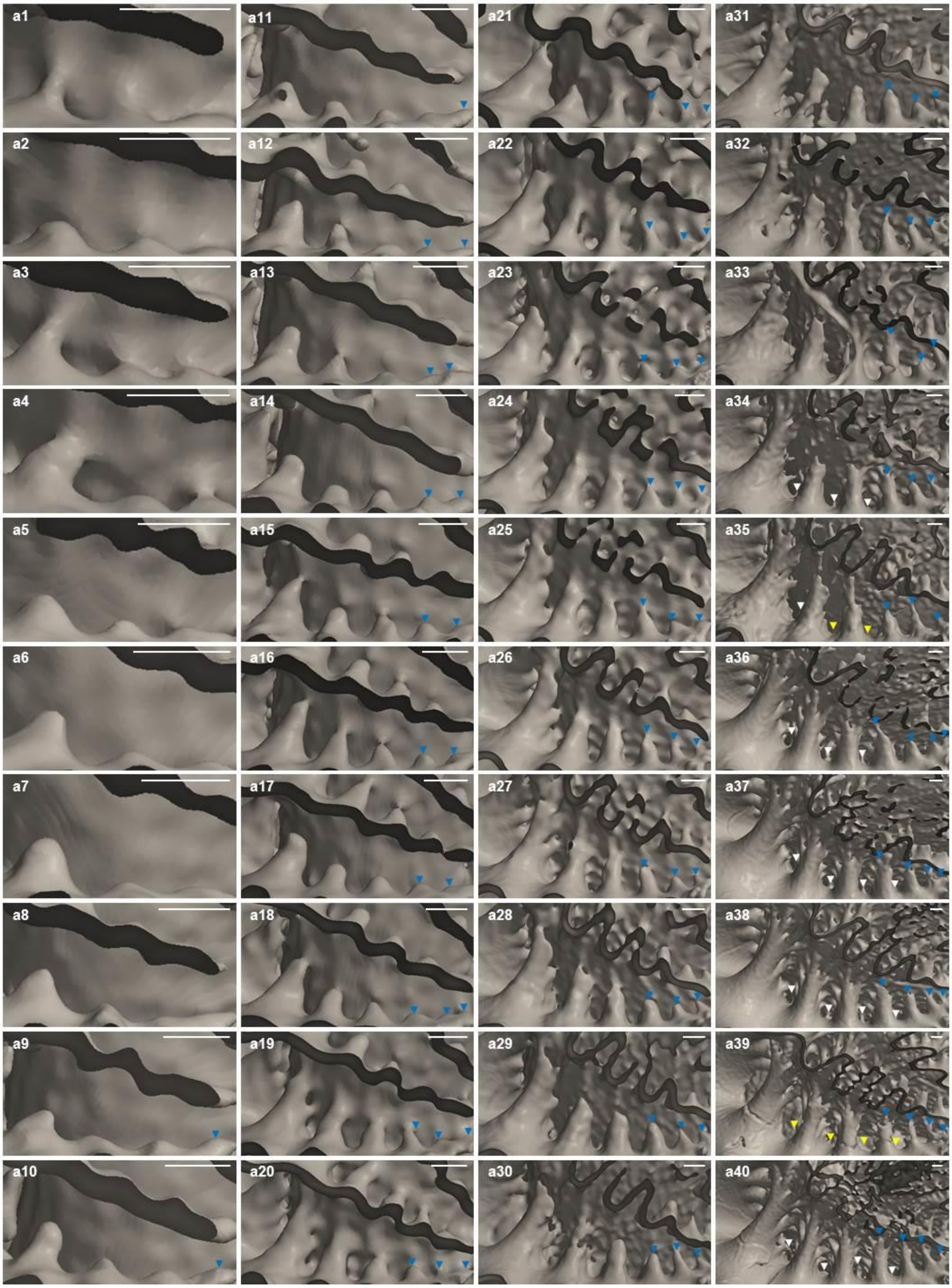

Supplementary Figure 4 | Three-dimensional data of forty consecutive umbilical arms of *Damesites* cf. *damesi* in the inner right regions. a1–40, New septal arms are indicated with arrowheads as in Figure 4. Blue and yellow arrowheads indicate the new septal arms at the distal site and hidden septal arms, respectively. Scale bars, 0.5 mm.

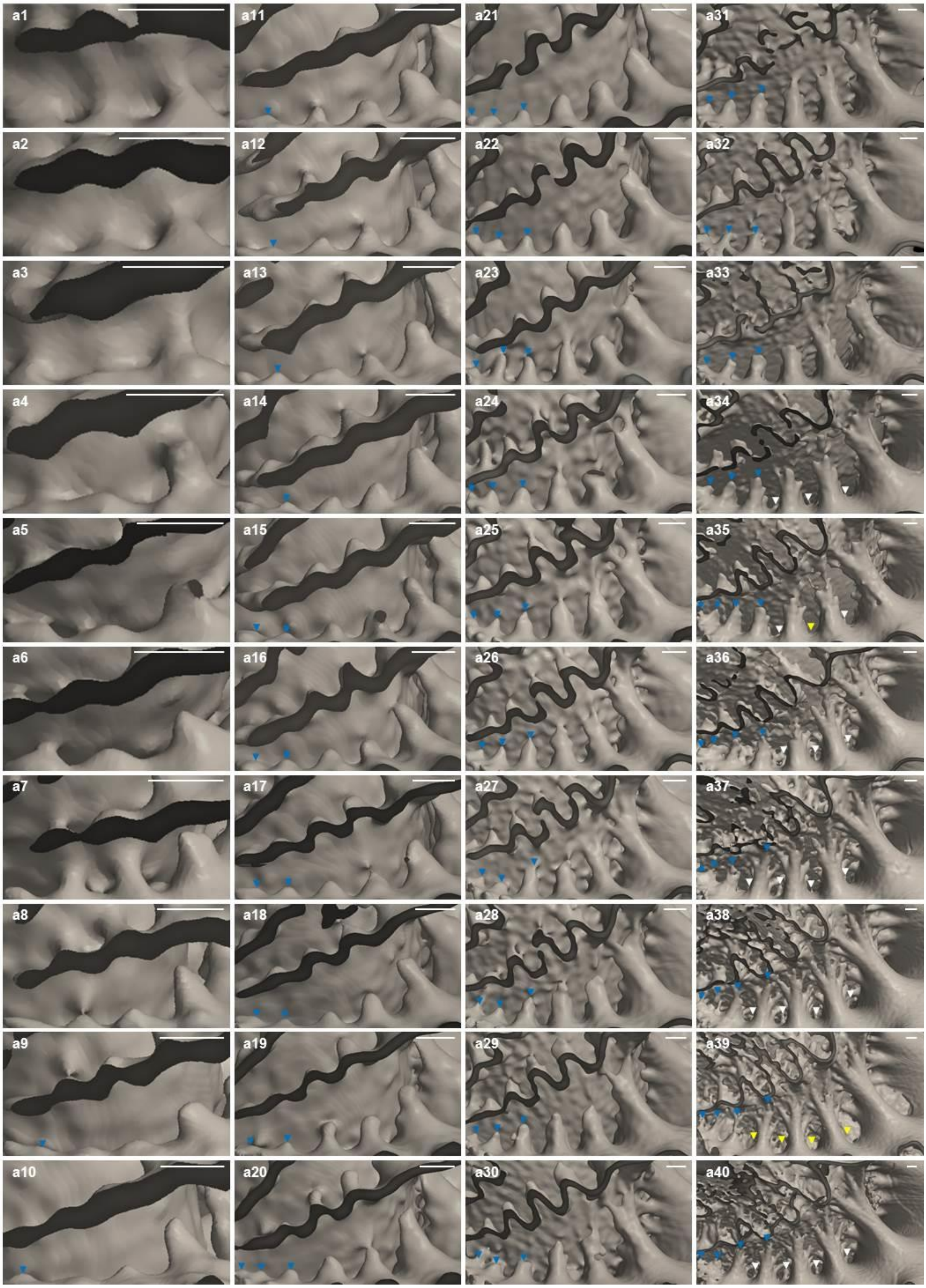

Supplementary Figure 5 | Three-dimensional data of forty consecutive umbilical arms of *Damesites* cf. *damesi* in the inner left regions. a1–40, New septal arms are indicated with arrowheads as in Figure 4. Blue and yellow arrowheads indicate the new septal arms at the

distal site and hidden septal arms, respectively. Scale bars, 0.5 mm.

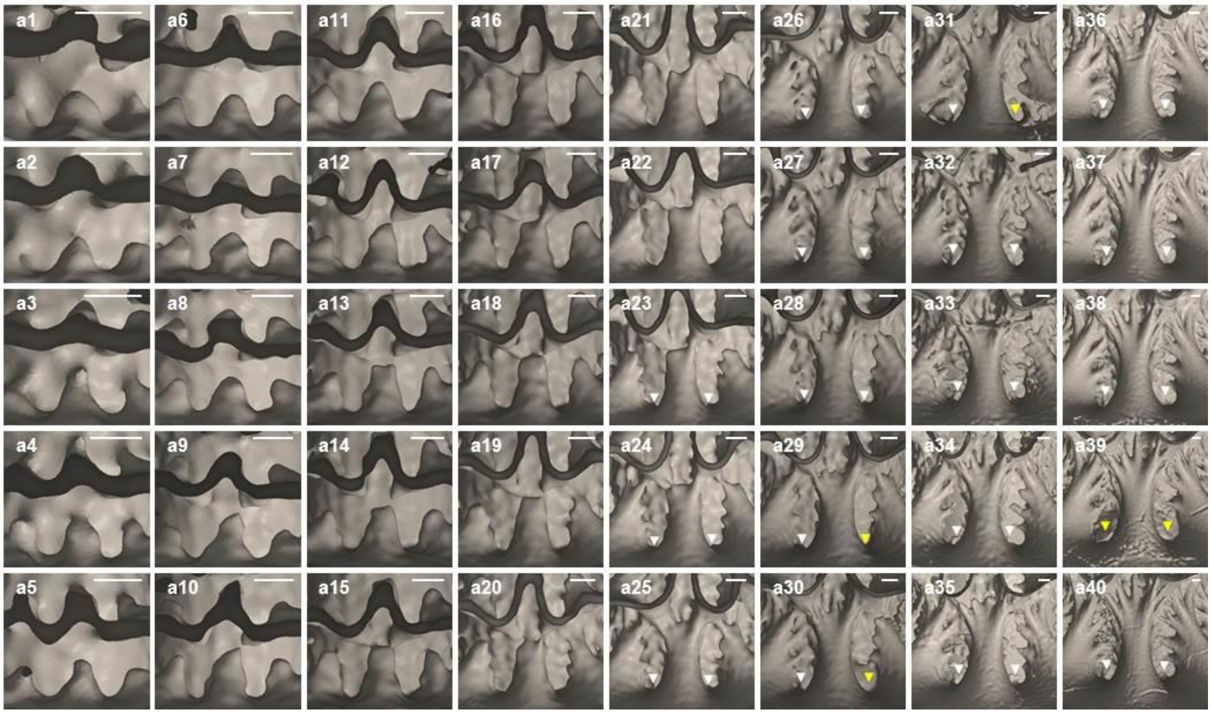

Supplementary Figure 6 | Three-dimensional data of forty consecutive internal arms of *Damesites* cf. *damesi*. a1–40, New septal arms are indicated with arrowheads as in Figure 4. Yellow arrowheads indicate hidden septal arms. Scale bars, 0.5 mm.

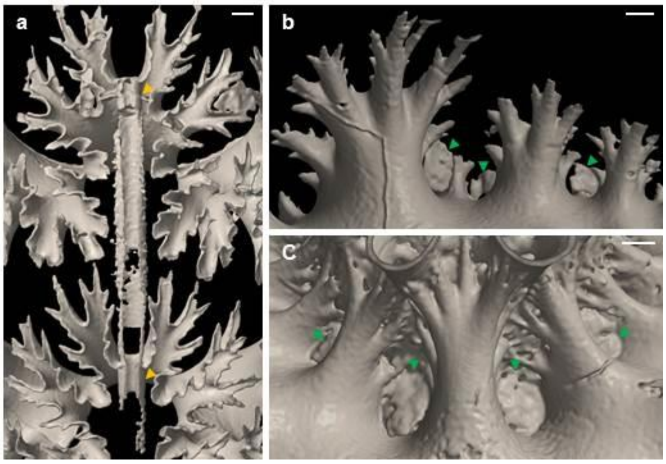

Supplementary Figure 7 | Three-dimensional data of septum-related structures. a, Ventral view of two septal necks and an intervening siphuncle. Orange arrowheads indicate septal necks. b, Cameral sheets in the outer region. c, Cameral sheets in the inner region. Green arrowheads indicate cameral sheets. Scale bars, 1 mm.

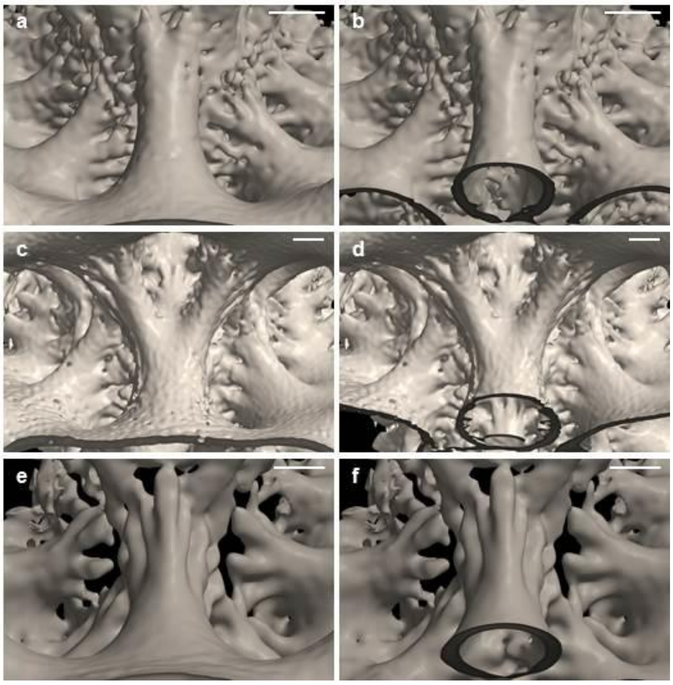

Supplementary Figure 8 | Tubular septal structures in the inner region of three suborders of ammonites. a, Inner region of the septum of NMA00811. b, Transverse section of the internal arm in a. c, Inner region of the septum of NMA00803. d, Transverse section of the internal arm in c. e, Inner region of the septum of NMA00812. f, Transverse section of the internal arm in e. Scale bars, 1 mm.

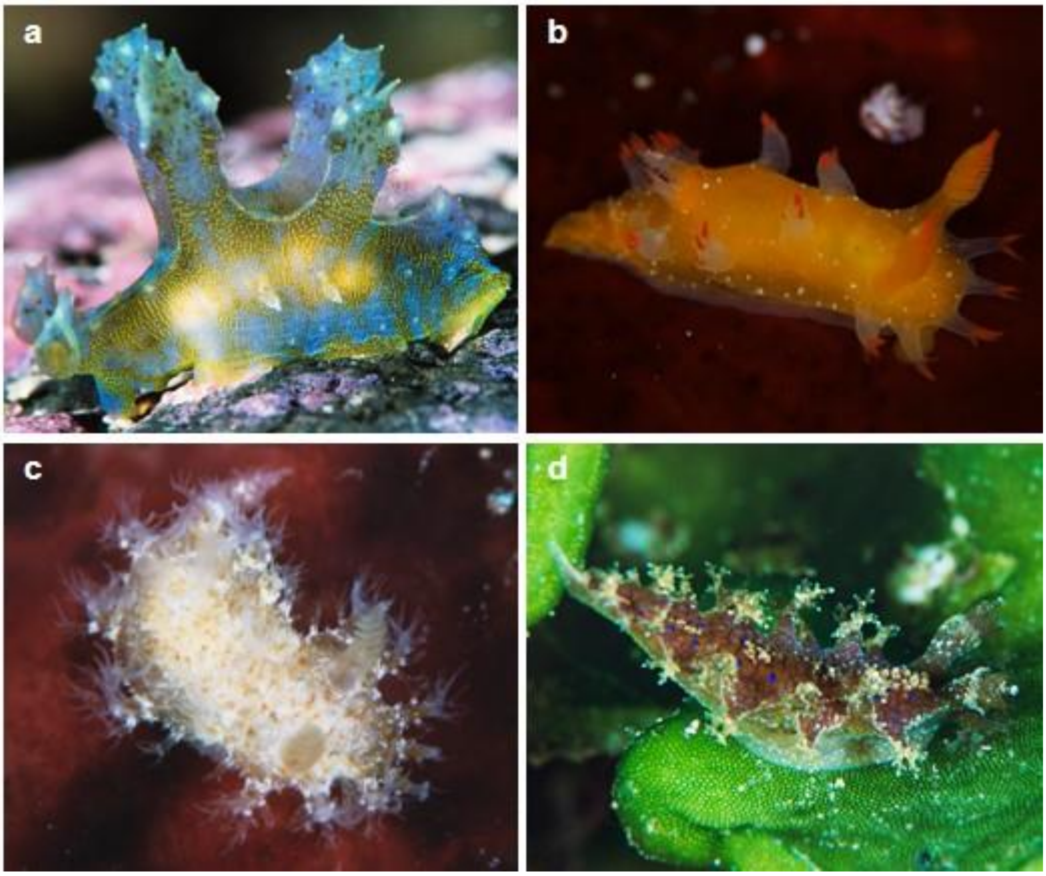

Supplementary Figure 9 | Various sea slugs with branched projections. a, *Scyllaea pelagica*. b, *Kaloplocamus acutus*. c, *Kaloplocamus* sp. d, *Marionia* sp (photographed by Kotaro Tanaka).

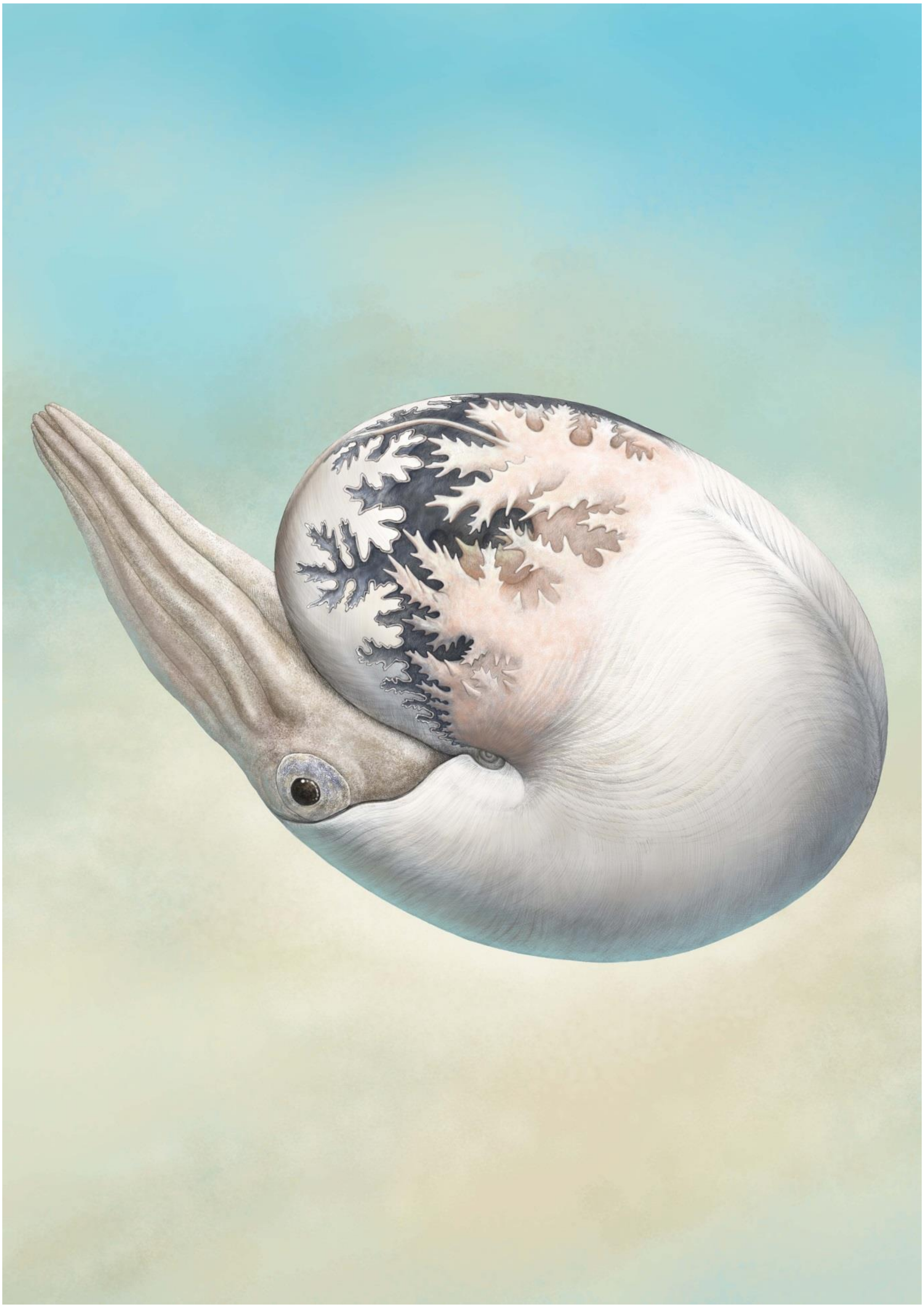

Supplementary Figure 10 | The conclusion of this paper, which is summarized by the imaginary drawing (illustrated by Takashi Oda).
